# Optimizing the Growth, Health, Reproductive Performance, and Gonadal Histology of Broodstock Fantail Goldfish (*Carassius auratus*, L.) by Dietary Cacao Bean Meal

**DOI:** 10.3390/ani10101808

**Published:** 2020-10-05

**Authors:** Hanan. S. Al-Khalaifah, Shimaa A. Amer, Dina M. M. Al-Sadek, Alshimaa A. Khalil, Eman M. Zaki, Doaa A. El-Araby

**Affiliations:** 1 Environment and Life Sciences Research Center, Kuwait Institute for Scientific Research, P.O. Box 24885, Safat 13109, Kuwait; hkhalifa@kisr.edu.kw; 2 Department of Nutrition and Clinical Nutrition, Faculty of Veterinary Medicine, Zagazig University, Zagazig 44511, Egypt; 3 Department of Histology and Cytology, Faculty of Veterinary Medicine, Zagazig University, Zagazig 44511, Egypt; dinaaalsadek@yahoo.com; 4 Department of Fish Diseases and Management, Faculty of Veterinary Medicine, Zagazig University, Zagazig 44511, Egypt; shvet2013@gmail.com; 5 Department of Reproductive Physiology and Hatchery, Central Laboratory for Aquaculture Research (CLAR), Agriculture Research Center, Abbassa 44662, Abo-Hammad, Sharkia, Egypt; dr.emanzaki1980@yahoo.com; 6 Department of Fish Health and Management, Central Laboratory for Aquaculture Research (CLAR), Agriculture Research Center, Abbassa 44662, Abo-Hammad, Sharkia, Egypt; dr.doaae@gmail.com

**Keywords:** fantail goldfish, growth performance, reproductive physiology, *Theobroma cacao* L.

## Abstract

**Simple Summary:**

Recently, the use of medicinal herbs for regulating reproduction has received much attention in aquaculture, as they are safe, effective, biodegradable, and locally available. The data on the use of cacao bean meal as a food supplement for fish are extremely scarce. This study assessed the possible effects of cacao bean meal as a feed supplement on the growth, health status, blood biochemical parameters, antioxidant, immune status, physiological parameters, female reproductive performance, and gonadal histological features of fantail goldfish. The experimental treatments consisted of three levels of cacao bean meal 0, 5, and 10 g kg^−1^ diet with the sex ratio being four females:two males per replicate. The findings suggested that cacao bean meal can be used as a feed supplement in diets of broodstock fantail goldfish for improving the growth, health status, and female reproductive performance, economic efficiency, and gonadal histological structure.

**Abstract:**

The potential effects of cacao bean meal, *Theobroma cacao* L., (CBM) on the growth, health status, blood biochemical parameters, antioxidant, immune status, physiological parameters, female reproductive performance, and gonadal histological features of fantail goldfish (*Carassius auratus*, L.) were evaluated using a complete randomized block design with sex as a block. The trial lasted for 60 days. A total of 54 healthy fantail goldfish (36 broodstock females and 18 broodstock males) were randomly allocated into three treatments with supplementation of three levels of cocoa powder 0, 5, and 10 g kg^−1^ diet, CBM0, CBM5, and CBM10, respectively, with the sex ratio being four females:two males per replicate. The body weight gain and feed conversion ratio of males were increased in the CBM10 treatment (*p* < 0.05). The CBM10 diet improved relative feed costs (*p* < 0.05). Females fed on the CBM10 diet had an increase in the serum level of total protein (*p* = 0.001). Females fed on a diet supplemented with CBM5 showed a decrease in the serum level of triglyceride compared to females fed on CBM0 and CBM10 diets (*p* = 0.03). CBM10 diet increased the serum superoxide dismutase (SOD) activity of fish compared to CBM0 and CBM5 diets (*p* = 0.004). Serum levels of testosterone and estradiol were significantly increased in males fed on the CBM10 diet. The female reproductive performance was improved by CBM supplementation (*p* < 0.05). Ovarian histology exhibited increased granulation and follicle numbers after dietary CBM supplementation compared to the control treatment. Therefore, cacao bean meal can be used as a feed supplement in the diets of fantail goldfish for improving the growth, health status, and female reproductive performance, economic efficiency, and gonadal histological structure.

## 1. Introduction

Goldfish (*Carassius auratus*, L.) is one of the most popular fish used as a biological model in many laboratories [1,2]. Goldfish may be an appropriate ideal fish for the study of reproductive performance due to its availability and the opportunity of obtaining a large number of eggs from one female fish [3,4]. Medicinal herbs significantly have been applied to the diets of various fish species as immunostimulants [5,6,7,8]. Furthermore, these herbs contain aromatic substances and essential oils used in the food industries [9]. Antimicrobial substances are now widely used for the treatment of bacterial diseases of fish [10,11]. Though, data about their effects on gonads and histological properties in fish is currently limited. The fish reproductive physiology is harmonized with nutritional, social, and environmental factors [12]. Herbal preparations aid to motivate gonadal maturation and increase the viability of eggs in female shrimp and spermatogenesis in male shrimp [13]. This improves egg quality and fertility and helps attain viable natural spawners particularly off-season. It was indicated that high levels of astaxanthin (150 mg kg^−1^) can improve the reproductive performance of broodstock goldfish [14]. Additionally, Tizkar, et al. [15] showed that astaxanthin supplementation (150 mg/kg) improved motility, osmolality, sperm concentration, and fertilization rate. In their recent study, Tizkar, et al. [16] found that dietary supplementation with higher levels of astaxanthin and β-carotene (150 mg kg^−1^) improved the gonadosomatic index in broodstock goldfish. The study of Kavitha, et al. [17] revealed that exposure of newly hatched sailfin molly *(Poecilia latipinna*) to *Tribulus terrestris* extract direct their sex more towards maleness and boosted spermatogenesis. On the other hand, a recent study reported a reduced absolute fecundity, gonadosomatic index, and bad changes in the gonadal histology of *Oreochromis niloticus,* which includes degenerating seminiferous tubules, few to absence of spermatozoa in the lumen of seminiferous tubules, and loss of the testicular architecture by using Aspilia plant (*Aspilia mossambicensis*) and Neem tree powders *(Azadirachta indica*) as feed supplement that was induced by alkali and flavonoids [18]. Another study indicated that dietary supplementation of *Carica papaya* extract could improve growth and enhance gonadal development in Nile tilapia [19]. 

Among all species of the *Theobroma genus*, *T. cacao* L. is only cultivated commercially and most visible in the market. *Theobroma cacao* is a herb that produces cocoa fruits and its raw beans are called cocoa. The cocoa seeds have been cultivated for a wide range of health benefits as a powerful antioxidant activity [20]. Cocoa extract enriched with polyphenols regulates the expression of various genes associated with oxidative stress, thus protecting the embryos of fish from induced oxidative stress. Cocoa extract triggered the activity of superoxide dismutase in embryos and tissues of adult fish, indicating a common mechanism of protection during embryonic development and maturity. Furthermore, cocoa extract feeding increased the life span of the fish [21]. Cocoa can improve blood flow and reduce cholesterol [22]. However, the flavonoids in cocoa have been shown to have promising anti-cancer properties in test-tube and animal studies [23]. No mortalities were reported from using cocoa meal in Nile tilapia diets up to 150 g kg^−1^ [24]. 

So this experiment was done for the first time to assess the potential effect of cacao bean meal (CBM) as a dietary supplement on the growth, health, and reproductive performance of fantail goldfish. 

## 2. Material and Methods

### 2.1. Fish and Rearing Condition 

The present study was conducted in one of Abu Sweir city farms, Ismailia Governorate, Egypt. The experiment was performed according to national and international institutional guidelines for the care and use of animals for scientific purposes, and ethical approval was obtained from the sponsoring institute Agriculture Research Center, Egypt (ARCIACUC-2019). The fish did not show any clinical abnormalities and did not have any history of disease outbreaks. The fish health status was checked before the experiment according to the guidelines of Canadian Council on Animal Care, CCAC [25].

A total of 54 healthy fantail goldfish (36 broodstock females with average bodyweight 58.6 ± 4.5 g and 18 broodstock males with average bodyweight 54.83 ± 3.18 g) were obtained from a private fish farm at Abu Sweir city, Ismailia Governorate, Egypt. From May to the middle of July, climatic conditions were suitable for spawning and the average water temperature was 25 ± 1 °C. The fish were kept in nine glass aquaria (60 L) capacity. About 25% of aquarium water (tap water free from chlorine) was exchanged daily. The aquaria were supplied with continuous aeration by air stone and fish were fed with a basal diet for two weeks before the beginning of the experiment. The water quality parameters were kept according to American Public Health Association, APHA [26] with a controlled photoperiod (12 h light: 12 h dark) in the laboratory. 

### 2.2. Preparation of Cacao Bean Meal, Diet Preparation, and Experimental Design 

Raw cacao beans were purchased from a local market (Zagazig city, Egypt). The beans were dried in a hot air oven at 105 °C for 3 h, crushed, and ground into a fine powder using an electrical blender, strained through a 0.25 mm sieve, and finally stored in the refrigerator (at 4 °C) in labeled airtight polyethylene bottles until its use. The proximate composition of cacao bean meal was measured according to AOAC [27], that revealed crude protein (20.3 ± 0.33%), ash (4.66 ± 0.20%), crude lipid (9.01 ± 0.23%), and moisture (8 ± 0.4%). 

Fish were randomly allocated into three treatments whose basal diets were supplemented with three levels of cacao bean meal 0, 5, and 10 g kg^−1^ diet, CBM0, CBM5, and CBM10, respectively, three replicates for each treatment (18 fish/treatment, 6 fish/replicate), with the sex ratio = four females:two males per replicate. The experiment lasted for 60 days. The proximate chemical composition of the basal diet (Table 1) was prepared to fulfill the recommended nutrient requirements of fish according to Gowsalya and Kumar [28]. The ingredients of the basal diet were mechanically mixed, pelletized, and air-dried at 27 °C for 24 h, following which the diets were stored at 4 °C in the refrigerator for further use. The fish were fed by hand till satiety three times daily (at 9:00 a.m. 12:00 a.m., and 4:00 p.m.) for 60 days. At the end of the feeding period, the collective feed intake (FI) of each treatment was determined and the fish were caught, sexed, weighed individually and the average final weights of males and females were recorded. The feed conversion ratio “FCR” was calculated as FCR = total feed intake (g)/total weight gain (g).

### 2.3. Gas Chromatography-Mass Spectrometry (GC-MS) Analysis of Cacao Bean Meal Extract

Cacao bean meal (50 g) was extracted using absolute ethanol for 6 h in a Soxhlet, and then the extract was filtered using Whatman filter paper No.1. After cooling the excess of solvents, aqueous and organic extract were removed under vacuum using a rotary evaporator. The extract was stored in the refrigerator (at 4 °C) for further use. The elements C, H, O, N, S were analyzed using the GC- mass techniques at the regional center for Mycology and Biotechnology Al-Azhar University, Egypt. The technique was performed using Trace GC1310-ISQ mass spectrometer (Thermo Scientific, Austin, TX, USA) with a direct capillary column TG–5MS (30 m × 0.25 mm × 0.25 µm film thickness). The column oven temperature was initially held at 50 C and then increased by 5 °C/min to 230 °C hold for 2 min, then increased to the final temperature of 290 °C by 30 °C/min and hold for 2 min. The injector and MS transfer line temperatures were kept at 250 and 260 °C respectively; helium was used as a carrier gas at a constant flow rate of 1 mL/min. The solvent delay was 3 min and diluted samples of 1 µl were injected automatically using an Autosampler AS1300 coupled with GC in the split mode. EI mass spectra were collected at 70 eV ionization voltages over the range of m/z 40–1000 in full scan mode. The ion source temperature was set at 200 °C. The components were identified by comparison of their retention times and mass spectra with those of WILEY 09 and NIST 11 mass spectral database.

Chromatographic characteristics by GC-mass techniques showing the active principles in cacao bean meal (Figure S1A); dodecanoic acid (lauric acid) (C12: 0) (area = 25.21%) (Figure S1B); tetradecanoic acid (myristic acid) (C14: 0) (area = 8.53%) (Figure S1C); hexadecanoic acid (palmitic acid) (C16: 0) (area = 8.29%) (Figure S2A); 9-Octadecenoic acid (oleic acid) (C18: 1n-9) (area = 5.85%) (Figure S2B); 9, 12-Octadecenoic acid (linoleic acid) (C18: 2n-6) (area = 5.59%) (Figure S2C); ascorbic acid 2,6-dihexadecanoate (C38H68O8) (area = 0.95%) (Figure S2D); desulphosinigrin (C10H17NO6S) (area = 0.21%) (Figure S3A); cholestan-3-ol, 2-methylene (area = 0.17%) (Figure S3B); melezitose (C18H32O16) (area = 0.16%) (Figure S3C); 1-heptatriacotanol (C37H76O) (area = 0.17%) (Figure S3D).

### 2.4. Economic Efficiency

Economic parameters were calculated according to El-Telbany and Atallah [30] and Dunning and Daniels [31], which include total costs, feed costs, and relative feed cost. 

Total costs (USD) = Fixed costs + feed costs. 

Feed costs (USD) = the cost of one kg of each diet including the cost of cacao bean meal (3.79 USD/kg) × the amount of total feed intake (kg) during the experimental period (60 days).

Relative feed cost (USD/kg of weight gain) = total feed costs/total weight gain

### 2.5. Blood Sampling

At the end of the experiment (60 days), ten fish/treatment (six females and four males) were collected and anesthetized with 100 mg L^−1^ benzocaine solution (Al-Nasr pharmaceutical chemicals Co, Egypt) according to Neiffer and Stamper [32], and blood samples were collected from the caudal blood vessels of fish by clean and sterile syringes without anticoagulant and the serum was separated by centrifuge at 1075× *g*. The obtained serum was used for the determination of some blood biochemical indices and the levels of the antioxidant enzymes. The serum was stored at −20 °C in screw cap glass vials until use [33].

The total protein, albumin was estimated following the technique described by Doumas, et al. [34]. The qualitative fractionation of serum proteins using cellulose-acetate electrophoresis was done according to Kaplan and Savory [35]. Serum triglycerides were estimated using colorimetric diagnostic kits of spectrum-bioscience (Egyptian Company for Biotechnology, Cairo, Egypt) following the method of McGowan, et al. [36].

Lysozyme activity was measured using fish Lysozyme ELISA kits with CAT. NO. MBS099538 following the instruction of the manufacturer (MyBioSource, Diego, USA). The activity of myeloperoxidase (MPO) was determined using fish Myeloperoxidase ELISA Kit (My Biosource Co. CAT NO. MBS016324). Serum nitric oxide (NO) was determined using Fish Inducible Nitric Oxide Synthase ELISA Kit (My Biosource Co. CAT NO. MBS023530).

Catalase (CAT) activity was measured using fish Catalase ELISA Kit (My Biosource Co. CAT NO. MBS038818). Superoxide dismutase (SOD) activity was evaluated using fish Superoxide Dismutase (SOD) ELISA kit (My Biosource Co. CAT NO. MBS705758). Reduced glutathione (GSH) level was estimated using reduced glutathione (GSH) Assay Kit (My Biosource Co. CAT NO. MBS2540412).

The serum level of follicle-stimulating hormone (FSH) was estimated using fish Follicle-Stimulating Hormone ELISA Kit (My Biosource Co. CAT NO. MBS281137). The serum level of Luteinizing Hormone (LH) was estimated using fish Luteinizing Hormone ELISA Kit (My Biosource Co. CAT NO. MBS031319). Testosterone hormone was assessed using fish Testosterone (T) ELISA Kit (My Biosource Co. CAT NO. MBS933475). 17 β estradiol hormone was measured using fish Estradiol ELISA Kit (My Biosource Co. CAT NO. MBS283228).

### 2.6. Reproductive Performance

The reproductive performance of female *C. auratus* was evaluated at two spawning periods; the first spawning was after 10 days from the beginning of the experiment and the second was after 25 days from the first. *Carassius auratus* eggs look like small round “bubbles”. They are clear in color except for a small dark spot in the middle of the egg. Healthy goldfish eggs look like small, clear bubbles and can range in color from white to yellow-orange. Dead eggs were pale yellow and were removed carefully. The eggs of C. auratus are adhesive and are attached to the roots of floating plants, which is best suited to the large aquarium and used as an egg collector. One of those plants that floated on the surface of the water was *Pistias tratiotes*, commonly called water lettuce. As soon as spawning was finished, the plants were transferred to a new aquarium with water temperature = 25 °C, where the eggs are hatched (72 h after spawning). Collection of seeds and swim-up fry first appeared during the 10 days of pairing. The seeds (fertile oval, newly hatched larvae with yolk sac and swim-up fry) were collected and counted after stocking up. The experiment was completed in 60 days.

The average number of eggs per spawning, the average number of fries per spawning, the average weight of eggs, the average weight of fry, embryonic development, and hatching rate of broodstock goldfish were calculated according to Boonyaratpalin [37].

Hatching rate% = no. of egg hatched/total no. of eggs × 100.

The average number of eggs per spawning = total number of eggs per tank/number of spawnings.

The average number of fries per spawning = total number of fries per tank/number of spawnings.

### 2.7. Histological Features

At the end of the experiment, 5 females and 3 males from each treatment were sacrificed by pithing, and separating the brain and spinal cord, and gonads were collected. Samples were fixed in 10% neutralized formalin solution, followed by washing with tap water, then dehydrated in ascending grades of ethyl alcohol (70–100%), cleared in xylene, and embedded in paraffin. Tissue sections of 5 µ thicknesses were prepared with the help of microtome (Leica^®^, Wetzlar, Germany) and stained with hematoxylin and eosin (H&E). Slides were examined and photographed using the AmScope digital camera-attached Ceti England microscope for histopathological examination [38]. 

### 2.8. Statistical Analysis

Shapiro–Wilk’s test was used to verify the normality and Levene’s test was used to verify homogeneity of variance components between experimental treatments and the assumption were achieved (*p* > 0.05). The data were analyzed using SPSS 18.0 for Windows (SPSS Inc., Chicago, IL, USA). Variations were assessed by complete randomized block design one-way (ANOVA) to examine the effect of cacao bean meal on the economic efficiency and female reproductive performance with controlling the effect of the spawning period. A complete randomized block design two-way (ANOVA) was used to examine the effect of cacao bean meal on the growth and blood biochemical parameters of fantail goldfish. Post-hoc Tukey’s multiple range tests were performed to compare the differences between the means at 5% probability. The variation in the data was expressed as the mean ± standard deviation (SD) and the significance level was set at *p* < 0.05. 

## 3. Results

### 3.1. Growth Performance:

The effect of CBM, sex, or their interaction on the growth performance parameters of *C. auratus* is shown in Table 2. The final body weight (FBW) and body weight gain (BWG) were significantly increased in the CBM10 diet compared to CBM0 and CBN5 diets (*p* = 0.001). The FCR was decreased in CBM10 diet compared to CBM0 diet (*p =* 0.01). Males showed higher FBW and BWG (*p =* 0.00) and lower FCR (*p =* 0.00) than females. The interaction between the CBM level and the sex increased the BWG in males fed the CBM10 diet compared to males fed CBM0 and CBM5 diets (*p =* 0.02). The BWG of males fed on CBM5 and CBM10 diets was increased by 10.21 and 22.06%, respectively. The collective feed intake (137.94 ± 0.66 g/fish) was not significantly affected by CBM supplementation (*p* > 0.05). 

### 3.2. Economic Efficiency

The economic value of using CBM as a feed supplement for *C. auratus* diets is shown in Table 3. The feed costs and total costs increased in the CBM10 diet in comparison with the CBM0 diet (*p* = 0.02). The relative feed cost was decreased in the CBM10 diet compared to the CBM0 diet (*p* = 0.02). The relative feed cost was improved by 5.08 and 14.88% for CBM5 and CBM10 diets, respectively. 

### 3.3. Serum Biochemical Parameters

The effect of CBM, sex, or their interaction on the blood biochemical parameters of *C. auratus* is shown in Table 4. CBM10 diet significantly increased the serum level of total protein of fish compared to CBM0 and CBM5 diets (*p =* 0.02). The serum levels of total protein, α2 globulin, and *β* globulin were significantly higher in males than in females (*p =* 0.00, *p =* 0.01, and *p =* 0.01, respectively). The interaction between the CBM level and the sex increased the serum level of total protein in females fed on the CBM10 diet compared to females fed on CBM0 and CBM5 diets (*p* = 0.001). Females fed on the CBM5 diet showed a significant decrease in the serum level of triglyceride compared to females fed on CBM0 and CBM10 diets (*p* = 0.03). 

Table 5 highlights the effect of CBM, sex, or their interaction on the antioxidant and immune status of *C. auratus.* CBM10 diet increased the serum SOD activity of fish compared to CBM0 and CBM5 diets (*p* = 0.004). *Carassius auratus* males had a higher serum level of GSH than females (*p =* 0.01). No significant effect of the interaction between the CBM level and the sex on the antioxidant and immune status of *C. auratus*. 

As shown in Table 6, the CBM10 diet significantly increased the serum level of FSH of fish compared to the CBM0 diet (*p =* 0.01). Dietary supplementation of CBM decreased the serum level of estradiol of fish compared to the CBM0 diet (*p* = 0.01). The serum levels of testosterone and estradiol were higher in males than in females (*p* = 0.00, *p* = 0.00, respectively). The interaction between the CBM level and the sex increased the serum levels of testosterone and estradiol in males fed on the CBM10 diet compared to males fed on the CBM0 diet (*p* = 0.03, *p =* 0.001, respectively).

### 3.4. Reproductive Performance

*Carassius auratus* eggs hatched from the CBM10 treatment first then eggs from the CBM5 treatment and finally eggs from the CBM0 treatment. The results concerning the effect of CBM on reproductive performance are illustrated in Table 7**.** The average egg weight, average fry weight, average number of eggs per spawning, average number of fries per spawning, and embryonic development were significantly increased in CBM5–10 treatments compared to CBM0 treatment (*p* = 0.000, *p* = 0.000, *p* = 0.000, *p* = 0.000, *p* = 0.001, respectively). The hatching rate% was significantly increased in CBM5 treatment compared to CBM0 treatment (*p =* 0.01). 

### 3.5. Histological Features

#### 3.5.1. Ovaries

**Figure 1:**
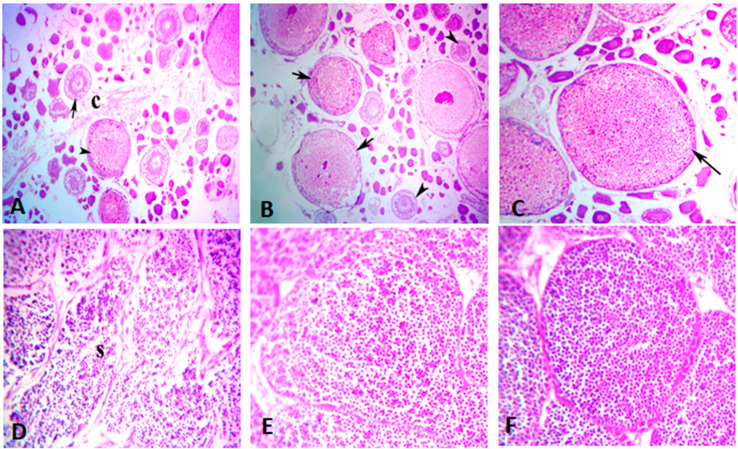
Photomicrographs of H&E-stained ovary (**A**–**C**) and testes (**D**–**F**) of *C. auratus,* fed cacao bean meal (CBM) for 60 days. (**A**) The control treatment showed normal ovary tissue structures with nearly normal previtellogenic follicles with or without oocytes (arrow), and limited maturation follicles that have yolk are deposited in the cytoplasm (arrowhead) surrounded by interstitial limit connective tissue (**C**) at 5×. (**B**) Fish fed CBM5 showed the normal structure of the mature stage of the oocyte (arrows) and different developmental stages of oocytes (arrowheads) at 10×. (**C**) CBM10 fed fish showed that the ovary becomes more developed and reached the final maturation (ripe oocytes) stage (arrow) at 10×. (**D**) Testes of fan goldfish fed CBM0 showed normal testicular seminiferous lobules engorged with spermatozoa (S), spermatogonia, and intra seminiferous lobules connective tissues at 10×. (**E**) Fish fed CBM5 diet showed histological maturation of testis with the normal structure of seminiferous tubules full of spermatocytes, spermatozoa, and spermatids at 10×. (**F**) Fish fed CBM10 showed the seminiferous tubules filled with sperms in the mature stage at 10×.

The ovaries of all the fish fed on CBM diets had the more or less similar architecture of the ovarian follicles, indicating that the supplementation of different levels of CBM was useful for the fish. Ovary of CBM0 treatment showed normal architecture of ovary with normal follicles and granulation (Figure 1A). CBM5-fed fish showed normal follicles with dense granulation (Figure 1B). CBM10-fed fish showed huge numbers of follicles with granulation (Figure 1C). The results exhibited increased granulation and numbers of follicles and better effect towards fecundity, granulation after the addition of various concentrations of CBM in the diet in comparison to control (Figure 1).

#### 3.5.2. Testes

Testes of fantail goldfish fed with CBM0 showed the normal structure of seminiferous tubules with spermatocytes, spermatids, and few sperms (Figure 1D). CBM5-fed fish showed histological maturation of testis with the normal structure of seminiferous tubules full of spermatocytes, spermatozoa, and spermatids (Figure 1E). In the case of fish fed with CBM10, testes showed the normal structure of seminiferous tubules full of spermatozoa and spermatids at the mature stage (Figure 1F).

## 4. Discussion

In aquaculture, the use of medicinal herbs to regulate reproduction has received great attention in recent times as they are safe, effective, biodegradable, and locally available. The data available for using cacao bean meal as a feed supplement for broodstock fantail goldfish is scarce. As an initial experiment, feeding of *C. auratus* with diets supplemented with cacao bean meal at a dose of 5 and 10 g kg^−1^ diet caused no mortalities among all treated groups, which help us to exclude the ID_50_. The results of the current work explained that diets supplemented with CBM could successfully boost some health parameters in *C. auratus*. The results indicated that CBM10-supplemented diets improved the growth performance of *C. auratus* and the interaction between the CBM level and the sex increased the BWG in males fed the CBM10 diet. Uzochukwu [39] reported an increased weight gain of mature African catfish, C*larias gariepinus,* fed on a diet supplemented with 10% CBM. The improved growth by CBM supplementation may be a result of the ascorbic acid content of cacao bean meal. L-ascorbic acid is a micronutrient important for normal physiological function and growth of most aquatic animals [40], as a result of the absence of L-gluconolactone oxidase that is responsible for the biosynthesis of ascorbic acid [41]. Adeebayo and Fawole [42] showed improved weight gain and FCR in broodstock African giant catfish, *Heterobranchus longifilis,* by dietary supplementation of ascorbic acid. The current study also showed that the body weight gain in males was higher than females, this may be because a considerable amount of energy in female fish is directed to reproductive activity and egg production as reported by [43].

Efforts to find ways to reduce the feed cost for fish production have evaluated a wide variety of feed supplements [6,44,45,46,47]. According the obtained results in the current study, the feed is the most expensive item in fish production, and it represents about 45% of total costs. Although CBM10 diet increased the feed costs, its positive effect on the fish growth reduced the relative feed costs compared to the control treatment. Therefore dietary CBM supplementation resulted in an improvement of economic efficiency. In contrast, Carvalho, et al. [24] reported an increase in the relative feed costs in diets supplemented with the cocoa meal compared to the control diet that can be attributed to the cost difference per kg of the diet so the economic analysis depends on the animal performance indexes. 

Regarding the effect of CBM, sex, or their interaction on the serum biochemical parameters, the interaction between CBM level and sex increased the serum level of total protein in females fed on the CBM10 diet and decreased the serum level of triglyceride in females fed on the CBM5 diet. This indicates the potential of CBM to increase defensive proteins, stimulating the immune response. The high values of blood proteins especially globulins are a good indicator of improved liver functions and innate immune response [48]. This may be attributed to the content of cacao bean meal extract from ascorbic acid. The results of Shahkar, et al. [49] showed that ascorbic acid supplementation can enhance the non-specific immune response of broodstock Japanese eel. Other studies reported hypotriglyceridemia and hypercholesteremic effects of polyphenol-rich foods such as cocoa products [50]. 

The oxidative stress can destroy many vital biological molecules such as DNA and proteins. There is an antioxidant defense system that protects the fish tissues from oxidative damages [51]. Antioxidant enzymes play a critical role in counteracting the oxidative stress caused by toxic substances [52,53] through scavenging peroxides and superoxide radicals. These enzymes are, for example, CAT that reduces hydrogen peroxide to water, SOD that converts superoxide anion radicals to hydrogen peroxide, GR that reduces oxidized glutathione to reduced glutathione GSH, and GPX that detoxifies hydrogen peroxide [54,55]. Regarding the effect of CBM supplementation on the antioxidant status of *C. auratus*, the CBM10 diet increased the serum SOD activity of fish and the males had a higher serum level of GSH than females, which could be attributed to the difference in the metabolic activity for both males and females [56]. The antioxidant properties of cacao bean meal can be attributed to the components of flavonoids that may prevent lipid peroxidation. The results of enhanced antioxidant activity can be attributed to cocoa polyphenols that are called flavanols, including epicatechins, catechins, and procyanidins which have strong antioxidant properties [57]. Flavanols and procyanidins exert physiological effects that include scavenging reactive oxygen species and preventing cellular oxidation [23]. Additionally, the GC-MS analysis of cacao bean meal extract indicates the presence of desulphosinigrin, cholestan-3-ol, 2-methylene, and 1-heptatriacotanol, which possess a potent antioxidant activity as reported by [58]. Beside, desulphosinigrin has an inhibitory activity on cyclin-dependent kinase 2 and contributed as a potent anticancer drug [59]. Additionally, melezitose is a sugar molecule, which possesses an anti-inflammatory effect that enhances fish health [60]. 

All vertebrates, including fish species, produce the gonadotropins (GH), luteinizing hormone (LH), and follicle-stimulating hormone (FSH) from the pituitary gland and they are the main regulators of gonadal reproduction and development. In fish, early phases of gametogenesis, such as spermatogenesis and vitellogenesis, are regulated by FSH, whereas the final maturation processes, such as ovulation, oocyte maturation, milt production, and sperms production, are the function of LH [61,62]. The effects of estradiol hormone (E2) on muscle protein turnover differ significantly between spermatozoa species [63]. Sexual maturation is the only period in the fish life cycle where there is a substantial rise of E2 levels which stimulate vitellogenin production from the liver [64]. So, the circulating levels of serum gonadotrophic hormones in fish have routinely been used as indicators for reproductive performance [65]. The results of the current study showed that dietary supplementation of CBM increased the serum level of FSH and the interaction between CBM level and sex increased the serum levels of testosterone and estradiol in males fed on the CBM10 diet. The mechanism by which the cacao bean meal affects the reproductive hormones is unknown but may be attributed to the content of cacao bean meal extract from ascorbic acid. Ascorbic acid contributes to the steroid hormones synthesis [66]. Dabrowski and Ciereszko [67] concluded that ascorbic acid is an important nutrient in reproductive tissue functions and high ascorbic acid concentrations have been linked to the tissues of gonads and brain in teleost fishes. They proposed that gonadal growth as a result of gonadotropin stimulation includes direct interaction between steroid hormones and catecholamines and their receptors. This interaction regulates absorption, transport, and metabolism of ascorbate in the reproductive system. Additionally, high concentrations of ascorbate fasten synthesis of adrenaline synthesis in neurohypophysis, which will lead to the improved secretion of gonadotropin hormones in the endocrine part. Ascorbic acid is a main constituent of steroidogenesis as it can be seen that its reduction in ovarian tissue is a vital way to measure luteinizing hormone (LH). Gonadotropin hormones motivate the synthesis of testosterone in the testes of fish [68]. On the other hand, steroid hormones such as estradiol (E2) promote GH synthesis in the pituitary gland, even though no release of blood circulation occurs [69]. For example, Seymour [70] reported a significant decrease in the ascorbic acid in goldfish ovaries as a result of the injection of the pituitary extract containing GH. In the current study, the serum levels of testosterone and estradiol were higher in males than in females, these changes could be related to the maturation of sexual tissues of both females and males and their reproductive cycle [71].

Female reproductive performance is strongly influenced by the nutritional status of fish that is well-known to have many reproductive traits, such as fecundity, age at maturity, egg size, embryonic development, and chemical composition of eggs [72,73,74]. The embryonic development of freshwater fish is influenced by the energetic reserves of the yolk sac. Previous research has shown that fry survival is influenced by yolk composition changes through the levels of diet and feeding [75,76]. The results of this study showed a positive effect of CBM supplementation on female reproductive performance. The improved reproductive performance by CBM supplementation may be attributed to the fatty acid composition of CBM from oleic acid and linoleic acid, which acts as energy-yielding substrates for reproduction. Dietary highly unsaturated fatty acids resulted in an increased number of fry production in female swordtail, *Xiphophorus helleri* [77]. Kolb, et al. [78] demonstrated that diets that contained higher levels of polyunsaturated fatty acids permitted broodstock Zebrafish (*Danio rerio*) to yield healthy larvae. Energy may be transferred from somatic growth during periods of increased energy demand, such as the end of the gonadal development [79,80], since the only source of fatty acids till the beginning of external feeding is maternal nutrition. 

Regarding the histological features of the gonads, the results exhibited increased granulation and numbers of follicles, and better effect towards fecundity and granulation after the addition of various concentrations of CBM. In the case of the testis, the histological features of all treatments were within the normal range. The study of Uzochukwu [39] indicated the highest ovarian weight and gonadosomatic index values in mature African catfish, *Clarias gariepinus,* females fed a diet supplemented with 10% CBM, and increased testicular weight and gonadosomatic index values in males fed a diet supplemented with 10 and 40% CBM. His results obtained for gonadal histology and histomorphology showed more developed gonads in fish fed diets supplemented with 10 and 40% CBM. The improved gonadal architecture in the current study can be attributed to the effect of CBM supplementation on boosting the serum gonadotrophic hormones and 17ß-estradiol hormone. Gonadotropin hormones (LH and FSH) are essential pituitary hormones that regulate the maturation and development of gonads [81,82,83]. FSH hormone seems to encourage the growth of new cells in the post-spawning period and transfer energy from somatic growth to the ovarian maturation [84]. The 17ß-estradiol has a significant role in fish reproductive physiology, particularly in the vitellogenesis process [85,86]. The 17ß-estradiol can accelerate the vitellogenin biosynthesis and the development of gonads [87]. Additionally, this improvement in the gonadal histology by CBM supplementation can be attributed to the ascorbic acid content of CBM, as reported in the study of Shahkar, et al. [49] who observed improved gonadal histology of broodstock Japanese eel, *Anguilla japonica,* indicated by increased spermatogonia number by dietary ascorbic acid addition.

## 5. Conclusions

The current study demonstrated that cacao bean meal can be used as a feed supplement in diets of fantail goldfish for improving the growth, health status, female reproductive performance, and gonadal histology of fantail goldfish. Additionally, supplementing the diets of fantail goldfish with cacao bean meal improves the relative feed cost; however, further studies are recommended to assess the inclusion of higher levels of CBM and to investigate the immunomodulation and other actions induced by CBM in different fish species.

## Figures and Tables

**Table 1:** Formulation and proximate composition of the experimental diets on air dry basis (g kg^−1^).

Item	CBM0	CBM5	CBM10
Fish meal 65% CP	100	100	100
Soybean meal 44% CP	431	431	431
Ground corn	163.1	163.1	163.1
Wheat bran	192.1	192.1	192.1
Wheat flour	40	35	30
Cacao bean meal	0	5	10
Fish oil	22.3	22.3	22.3
Corn oil	16.5	16.5	16.5
Methionine	5	5	5
Vitamins premix ^A^	10	10	10
Minerals Premix ^B^	20	20	20
Proximate chemical analysis (g kg^−1^)			
Dry matter	916.80	914.60	915.30
Crude protein	301.00	301.40	301.90
Crude fat	59.70	60.00	60.40
Ash	8.13	8.33	8.17
Crude fiber	58.02	57.60	57.50
Nitrogen free extract	489.95	487.27	487.33
Lysine	18.60	18.50	18.40
Methionine	9.82	9.81	9.80
DE (Kcal/kg) *	2553	2556	2558

^A^ Vitamin premix (per kg of premix): thiamine, 2.5 g; riboflavin, 2.5 g; pyridoxine, 2.0 g; inositol, 100.0 g; biotin, 0.3 g; pantothenic acid, 100.0 g; folic acid, 0.75g; para-aminobenzoic acid, 2.5 g; choline, 200.0 g; nicotinic acetate, 10.0 g; menadione, 500.000 IU. ^B^ Mineral premix (g/kg of premix): Dicalcium phosphate (CaHPO4.2H2O), 727.2; magnesium sulfate heptahydrate (MgCO4.7H2O), 127.5; potassium chloride (KCI) 50.5; sodium chloride (NaCI), 60.0; ferric citrate trihydrate (FeC6H5O7.3H2O), 25.0; zinc carbonate (ZnCO3), 5.5; manganese chloride tetrahydrate (MnCL2.4H2O), 2.5; copper acetate monohydrate (CU(OAc)2. H2O), 0.785; cobalt chloride hexahydrate (CoCL3.6H2O), 0.128; aluminum chloride hexahydrate (AICI3.6 H2O), 0.477; chromium chloride hexahydrate (CrCI3.6 H2O), 0.128; sodium selenite (Na2SeO3), 0.03. * Digestible energy (DE) was calculated based on values of protein 3.5 Kcal g ^−1^, fat 8.1 Kcal g ^−1^, NFE 2.5 Kcal g ^−1^ according to Santiago, et al. [29]. CP: crude protein

**Table 2:** Effect of dietary supplementation of cacao bean meal (CBM) on the growth performance of broodstock *C. auratus*.

Item	IBW(g/Fish)	FBW(g/Fish)	BWG (g/Fish)	FCR
CBM level (g kg^−1^ diet)				
0	56.50 ± 2.25	105.17 ± 1.56 ^b^	48.67 ± 4.25 ^b^	3.01 ± 0.04 ^a^
5	57.25 ± 3.35	110.21 ± 2.65 ^b^	52.96 ± 3.65 ^b^	2.83 ± 0.03 ^ab^
10	56.39 ± 4.65	119.57 ± 5.26 ^a^	63.17 ± 2.24 ^a^	2.46 ± 0.07 ^b^
*p-*Value	0.94	0.00	0.00	0.01
Sex				
Male	54.83 ± 3.24	125.70 ± 2.25 ^a^	70.86 ± 3.26 ^a^	1.97 ± 0.01 ^b^
Female	58.59 ± 6.35	97.60 ± 2.36 ^b^	39.01 ± 1.25 ^b^	3.56 ± 0.04 ^a^
*p-*Value	0.16	0.00	0.00	0.00
Interaction				
CBM0 × Male	55.00 ± 4.24	116.67 ± 1.51	61.67 ± 2.72 ^b^	2.21 ± 0.06
CBM5 × Male	55.50 ± 3.53	123.47 ± 2.08	67.97 ± 1.45 ^b^	2.03 ± 0.04
CBM10 × Male	54.00 ± 4.24	136.97 ± 0.59	82.97 ± 3.64 ^a^	1.69 ± 0.07
CBM0 × Female	58.00 ± 4.35	93.68 ± 6.18	35.68 ± 1.93 ^c^	3.81 ± 0.15
CBM5 × Female	59.00 ± 4.24	96.97 ± 0.81	37.97 ± 3.42 ^c^	3.65 ± 0.40
CBM10 × Female	58.79 ± 3.93	102.17 ± 4.26	43.39 ± 0.32 ^c^	3.23 ± 0.01
*p-*Value	0.95	0.10	0.02	0.94

^a, b, c^ Means within the same row carrying different superscripts are significantly different at (*p* < 0.05) according to post-hoc Tukey’s multiple range tests. CBM0—control diet (with no additives), CBM5—basal diet supplemented with cacao bean meal 5 g kg^−1^ diet, CBM10—basal diet supplemented with cacao bean meal 10 g kg^−1^ diet, IBW—initial body weight, FBW—final body weight, BWG—body weight gain, FI—feed intake, FCR—feed conversion ratio.

**Table 3:** Effect of dietary supplementation of cacao bean meal on economic efficiency.

Item	Feed Costs (USD)	Total Costs (USD)	Relative Feed Cost (USD/kg WG)
CBM0	0.53 ± 0.007 ^b^	1.16 ± 0.007 ^b^	1.98 ± 0.04 ^a^
CBM5	0.54 ± 0.02 ^b^	1.17 ± 0.02 ^b^	1.88 ± 0.07 ^ab^
CBM10	0.57 ± 0.002 ^a^	1.21 ± 0.002 ^a^	1.69 ± 0.03 ^b^
*p-*Value	0.02	0.02	0.02

^a, b^ Means within the same row carrying different superscripts are significantly different at (*p* < 0.05) according to post-hoc Tukey’s multiple range tests. Variable costs (USD) = feed cost (the cost of one kg of each diet including the cost of cacao bean meal (3.79 USD/kg) × the amount of total feed intake (kg) during the experimental period (60 days). The cost of each kg diet was 0.647, 0.654, and 0.681 USD for CBM0, CBM5, and CBM10, respectively. CBM0—control diet (with no additives), CBM5—basal diet supplemented with cacao bean meal 5 g kg^−1^ diet, CBM10—basal diet supplemented with cacao bean meal 10 g kg^−1^ diet.

**Table 4:** Effect of dietary supplementation of cacao bean meal (CBM) on the blood biochemical parameters of broodstock *C. auratus*.

Item	TG (g/dL)	TP (g/dL)	Albumin (g/dL)	α1 Globulin (g/dL)	α2 Globulin (g/dL)	β Globulin (g/dL)	γ Globulin (g/dL)
CBM level (g kg^−1^ diet)							
0	2.81 ± 0.04	7.34 ± 0.08 ^ab^	4.52 ± 0.12	1.3 ± 0.07	0.5 ± 0.02	0.61 ± 0.19	0.4 ± 0.01
5	2.59 ± 0.03	7.28 ± 0.06 ^b^	4.67 ± 0.16	1.02 ± 0.06	0.46 ± 0.01	0.6 ± 0.11	0.53 ± 0.05
10	2.67 ± 0.01	7.45 ± 0.03 ^a^	4.78 ± 0.09	1.3 ± 0.08	0.48 ± 0.03	0.62 ± 0.09	0.28 ± 0.03
*p-*Value	0.88	0.02	0.86	0.49	0.81	0.97	0.15
Sex							
Male	3.13 ± 0.08	7.53 ± 0.01 ^a^	4.39 ± 0.13	1.35 ± 0.002	0.57 ± 0.01 ^a^	0.76 ± 0.23 ^a^	0.46 ± 0.11
Female	2.25 ± 0.06	7.19 ± 0.03 ^b^	4.92 ± 0.08	1.06 ± 0.03	0.39 ± 0.02 ^b^	0.46 ± 0.12 ^b^	0.34 ± 0.15
*p-*Value	0.05	0.00	0.21	0.22	0.01	0.01	0.23
Interaction							
CBM0 × Male	3.49 ± 0.51 ^a^	7.60 ± 0.08 ^a^	4.11 ± 0.43	1.55 ± 0.21	0.62 ± 0.02 ^a^	0.87 ± 0.05 ^a^	0.45 ± 0.33
CBM5 × Male	3.71 ± 0.12 ^a^	7.58 ± 0.07 ^a^	3.86 ± 0.04	1.44 ± 0.07	0.65 ± 0.02 ^a^	0.86 ± 0.01 ^a^	0.76 ± 0.01
CBM10 × Male	2.19 ± 0.96 ^ab^	7.42 ± 0.09 ^a^	5.22 ± 0.06	1.06 ± 0.8	0.45 ± 0.15 ^ab^	0.55 ± 0.35 ^ab^	0.18 ± 0.01
CBM0 × Female	2.14 ± 0.06 ^ab^	7.00 ± 0.02 ^b^	5.48 ± 0.09	1.05 ± 0.07	0.38 ± 0.02 ^ab^	0.35 ± 0.05 ^b^	0.35 ± 0.08
CBM5 × Female	1.48 ± 0.04 ^c^	6.99 ± 0.01 ^b^	5.56 ± 0.11	0.60 ± 0.007	0.29 ± 0.02 ^b^	0.34 ± 0.05 ^b^	0.31 ± 0.06
CBM10 × Female	3.15 ± 0.05 ^a^	7.50 ± 0.03 ^a^	4.34 ± 0.10	1.55 ± 0.77	0.51 ± 0.12 ^ab^	0.70 ± 0.15 ^ab^	0.39 ± 0.11
*p-*Value	0.03	0.00	0.08	0.09	0.02	0.04	0.05

^a, b, c^ Means within the same row carrying different superscripts are significantly different at (*p* < 0.05) according to post-hoc Tukey’s multiple range tests. CBM0—control diet (with no additives), CBM5—basal diet supplemented with cacao bean meal 5 g kg^−1^ diet, CBM10—basal diet supplemented with cacao bean meal 10 g kg^−1^ diet, TG—triglycerides, TP—total protein.

**Table 5:** Effect of dietary supplementation of cacao bean meal (CBM) on the antioxidant and immune status of broodstock *C. auratus*.

Item	CAT (U/L)	SOD (U/mL)	GSH (mmol/L)	MPO (U/L)	NO (μmol/L)	Lysozyme (U/L)
CBM level (g kg^−1^ diet)						
0	112.75 ± 7.25	3.35 ± 0.26 ^b^	1.15 ± 0.01	5.74 ± 0.12	20.00 ± 3.25	22.25 ± 3.20
5	101.50 ± 9.25	3.87 ± 0.45 ^b^	0.95 ± 0.03	6.01 ± 0.13	11.75 ± 2.45	21.5 ± 4.56
10	138.25 ± 10.35	6.06 ± 0.25 ^a^	1.64 ± 0.04	5.96 ± 0.23	19.75 ± 4.12	30.00 ± 2.25
*p-*Value	0.10	0.00	0.17	0.93	0.28	0.05
Sex						
Male	126 ± 3.26	4.53 ± 0.13	1.68 ± 0.02 ^a^	6.03 ± 0.26	17.50 ± 4.56	27.16 ± 1.23
Female	109 ± 2.55	4.33 ± 0.26	0.82 ± 0.05 ^b^	5.77 ± 0.12	16.83 ± 3.35	22 ± 3.25
*p-*Value	0.20	0.65	0.01	0.70	0.88	0.07
Interaction						
CBM0 × Male	104.00 ± 5.65	3.22 ± 0.19	1.51 ± 0.01	5.82 ± 0.95	18.50 ± 3.53	21.00 ± 5.65
CBM5 × Male	127.00 ± 6.66	4.00 ± 0.141	1.63 ± 0.78	7.02 ± 0.16	16.00 ± 4.24	25.00 ± 4.24
CBM10 × Male	147.00 ± 14.14	6.365 ± 1.025	1.90 ± 0.35	5.26 ± 1.66	18.00 ± 2.82	35.50 ± 4.95
CBM0 × Female	121.50 ± 10.6	3.49 ± 0.83	0.80 ± 0.17	5.66 ± 1.89	21.50 ± 10.60	23.50 ± 3.53
CBM5 × Female	76.00 ± 5.65	3.76 ± 1.05	0.28 ± 0.03	5.00 ± 0.02	7.50 ± 3.56	18.00 ± 2.82
CBM10 × Female	129.50 ± 6.36	5.76 ± 0.46	1.39 ± 0.70	6.67 ± 0.24	21.50 ± 13.43	24.50 ± 3.53
*p-*Value	0.14	0.70	0.44	0.17	0.49	0.14

^a, b^ Means within the same row carrying different superscripts are significantly different at (*p* < 0.05) according to post-hoc Tukey’s multiple range tests. CBM0—control diet (with no additives), CBM5—basal diet supplemented with cacao bean meal 5 g kg^−1^ diet, CBM10—basal diet supplemented with cacao bean meal 10 g kg^−1^ diet, CAT—catalase, SOD—superoxide dismutase, GSH—reduced glutathione, NO—nitric oxide, MPO—myeloperoxidase.

**Table 6:** Effect of dietary supplementation of cacao bean meal (CBM) on the serum levels of reproductive hormones of broodstock *C. auratus*.

Item	FSH (mIU/mL)	LH (mIU/mL)	TES (ng/mL)	E2 (pg/mL)
CBM level (g kg^−1^ diet)				
0	0.16 ± 0.02 ^b^	1.34 ± 0.02	0.54 ± 0.04	1147.75 ± 15.04 ^a^
5	0.23 ± 0.01 ^a^^b^	1.14 ± 0.01	0.58 ± 0.03	946.7 ± 20.04 ^b^
10	0.36 ± 0.03 ^a^	1.54 ± 0.03	0.61 ± 0.01	939.85 ± 8.02 ^b^
*p-*Value	0.01	0.36	0.09	0.01
Sex				
Male	0.27 ± 0.01	1.51 ± 0.01	0.83 ± 0.03 ^a^	1232.23 ± 17.25 ^a^
Female	0.22 ± 0.02	1.17 ± 0.02	0.33 ± 0.01 ^b^	790.63 ± 3.5 ^b^
*p-*Value	0.24	0.16	0.00	0.00
Interaction				
CBM0 × Male	0.19 ± 0.01	1.47 ± 0.04	0.75 ± 0.06 ^b^	1152.20 ^±^ 6.77 ^b^
CBM5 × Male	0.25 ± 0.09	1.51 ± 0.12	0.83 ± 0.02 ^ab^	1183.80 ± 15.77 ^ab^
CBM10 × Male	0.37 ± 0.10	1.56 ± 0.05	0.91 ± 0.02 ^a^	1360.60 ± 8.98 ^a^
CBM0 × Female	0.13 ± 0.04	1.22 ± 0.02	0.34 ± 0.01 ^c^	1143.30 ± 7.94 ^c^
CBM5 × Female	0.21 ± 0.01	1.41 ± 0.89	0.33 ± 0.02 ^c^	709.55 ± 1.34 ^c^
CBM10 × Female	0.34 ± 0.03	1.54 ± 0.04	0.32 ± 0.02 ^c^	519.05 ± 3.60 ^c^
*p-*Value	0.94	0.43	0.03	0.00

^a, b, c^ Means within the same row carrying different superscripts are significantly different at (*p* < 0.05) according to post-hoc Tukey’s multiple range tests. CBM0—control diet (with no additives), CBM5—basal diet supplemented with cacao bean meal 5 g kg^−1^ diet, CBM10—basal diet supplemented with cacao bean meal 10 g kg^−1^ diet. Follicle-stimulating hormone (FSH), luteinizing hormone (LH), Testosterone (TES), and estradiol (E2).

**Table 7:** Effect of dietary supplementation of cacao bean meal (CBM) on the reproductive performance of female broodstock *C. auratus*.

Item	Average Egg Wt. (g)	Average Fry Wt. (g)	Eggs/Spawning	Fries/Spawning	Hatching Rate%	Embryonic Development
CBM0	22.50 ± 2.16 ^b^	5.83 ± 0.32 ^b^	3281.17 ± 390.06 ^b^	2383.67 ± 242.52 ^b^	72.83 ± 5.03 ^b^	5.17 ± 0.40 ^b^
CBM5	34.50 ± 3.78 ^a^	9.00 ± 0.63 ^a^	5210.67 ± 514.07 ^a^	4327.83 ± 546.96 ^a^	83.00 ± 5.17 ^a^	5.83 ± 0.42 ^a^
CBM10	35.00 ± 3.22 ^a^	8.83 ± 0.75 ^a^	5321.33 ± 484.61 ^a^	4257.67 ± 315.97 ^a^	80.33 ± 6.91 ^ab^	6.00 ± 0.20 ^a^
*p-*Value	0.00	0.00	0.00	0.00	0.01	0.001

^a, b^ Means within the same row carrying different superscripts are significantly different at (*p* < 0.05) according to post-hoc Tukey’s multiple range tests. CBM0—control diet (with no additives), CBM5—basal diet supplemented with cacao bean meal 5 g kg^−1^ diet, CBM10—basal diet supplemented with cacao bean meal 10 g kg^−1^ diet.

## Supplementary Materials

The following are available online at https://www.mdpi.com/2076-2615/10/10/1808/s1, Figure S1. (A) Chromatographic characteristics by GC-mass techniques showing the active principles in cacao bean meal. (B) Dodecanoic acid (lauric acid) (C12: 0) (area= 25.21%). (C) Tetradecanoic acid (myristic acid) (C14: 0) (area = 8.53%). Figure S2. Chromatographic characteristics by GC-mass techniques showing the active principles in cacao bean meal. (A) Hexadecanoic acid (palmitic acid) (C16: 0) (area = 8.29%). (B) 9-Octadecenoic acid (oleic acid) (C18: 1n-9) (area = 5.85%). (C) 9,12-Octadecenoic acid (linoleic acid) (C18: 2n-6) (area = 5.59%). (D) Ascorbic acid 2,6-dihexadecanoate (C38H68O8) (area = 0.95%). Figure S3. Chromatographic characteristics by GC-mass techniques showing the active principles in cacao bean meal. (A) Desulphosinigrin (C10H17NO6S) (area = 0.21%). (B) Cholestan-3-ol, 2-methylene (area = 0.17%). (C) Melezitose (C18H32O16) (area = 0.16%). (D) 1-Heptatriacotanol (C37H76O) (area = 0.17%).
