# Supplementary material for: Optimizing the Growth, Health, Reproductive Performance, and Gonadal Histology of Broodstock Fantail Goldfish (Carassius auratus, L.) by Dietary Cacao Bean Meal
Source: Animals (Basel). 2020 Oct 5;10(10):1808. doi: 10.3390/ani10101808 (PMC7600488; doi:10.3390/ani10101808)
Supplement: Supplementary file 1 [file animals-10-01808-s001.pdf]

animals

Supplementary Materials: Optimizing the Growth, Health, Reproductive Performance, and Gonadal Histology of Broodstock Fantail Goldfish (*Carassius auratus*, L.) by Dietary Cacao Bean Meal

Hanan. S. Al-Khalaifah [1], Shimaa A. Amer [2,*], Dina M.M. Al-Sadek [3], Alshimaa A. Khalil [4], Eman M. Zaki [5] and Doaa A. El-Araby [6]

[1] Environment and Life Sciences Research Center, Kuwait Institute for Scientific Research, P.O. Box 24885, Safat 13109, Kuwait; hkhalifa@kisr.edu.kw
[2] Department of Nutrition and Clinical Nutrition, Faculty of Veterinary Medicine, Zagazig University, 44511 Zagazig, Egypt
[3] Department of Histology and Cytology, Faculty of Veterinary Medicine, Zagazig University, 44511 Zagazig, Egypt.; dinaaalsadek@yahoo.com
[4] Department of Fish Diseases and Management, Faculty of Veterinary Medicine, Zagazig University, 44511 Zagazig , Egypt; shvet2013@gmail.com
[5] Department of Reproductive Physiology and Hatchery, Central Laboratory for Aquaculture Research (CLAR), Agriculture Research Center, Abbassa, 44662 Abo-Hammad, Sharkia, Egypt; dr.emanzaki1980@yahoo.com
[6] Department of Fish Health and Management, Central Laboratory for Aquaculture Research (CLAR), Agriculture Research Center, Abbassa, 44662 Abo-Hammad, Sharkia, Egypt; dr.doaae@gmail.com
* Correspondence: shimaa.amer@zu.edu.eg

Received: 14 August 2020; Accepted: 24 September 2020; Published: date

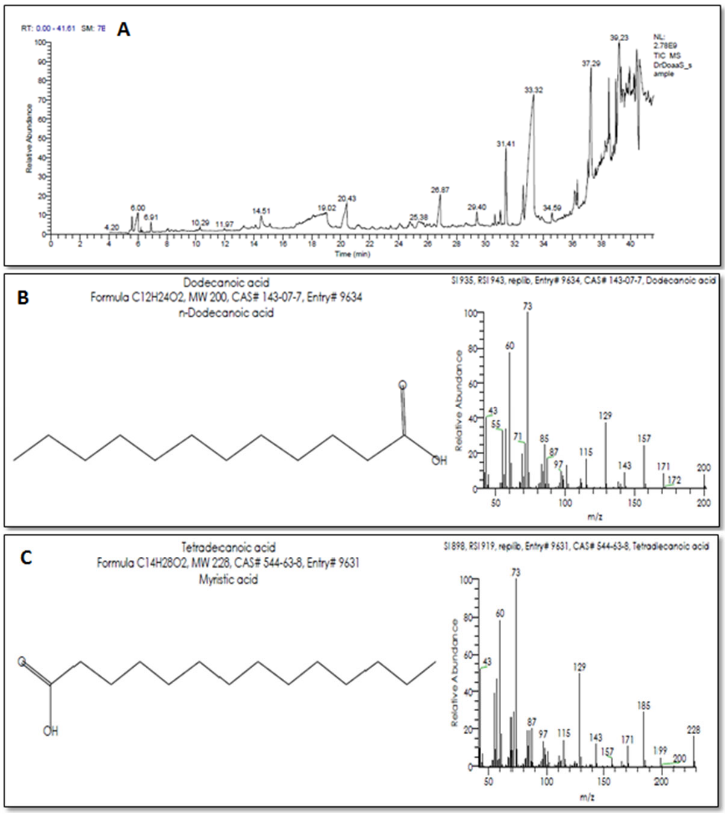

Figure S1. (**A**) Chromatographic characteristics by GC-mass techniques showing the active principles in cacao bean meal. (**B**) Dodecanoic acid (lauric acid) (C12: 0) (area= 25.21%). (**C**) Tetradecanoic acid (myristic acid) (C14: 0) (area = 8.53%).

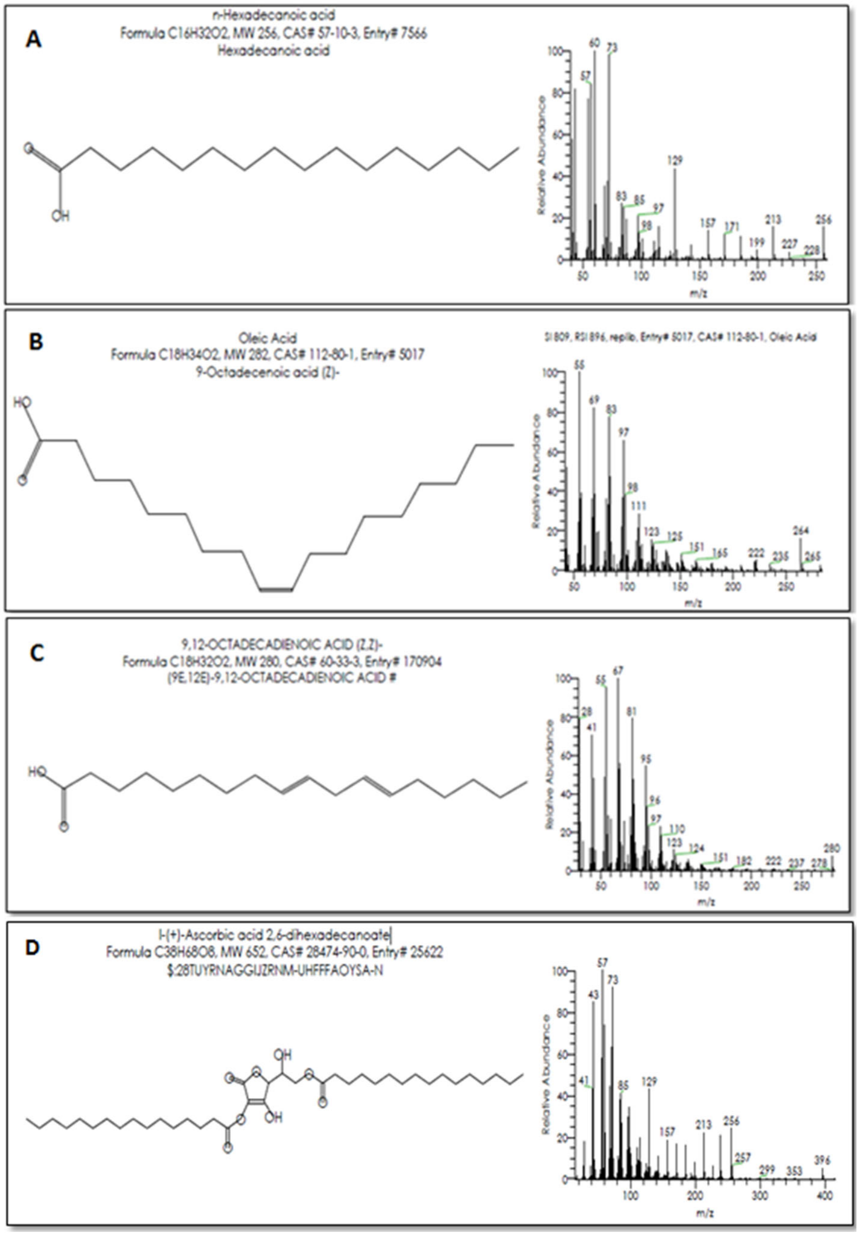

Figure S2. Chromatographic characteristics by GC-mass techniques showing the active principles in cacao bean meal. (**A**) Hexadecanoic acid (palmitic acid) (C16: 0) (area = 8.29%). (**B**) 9-Octadecenoic acid (oleic acid) (C18: 1n-9) (area = 5.85%). (**C**) 9,12-Octadecenoic acid (linoleic acid) (C18: 2n-6) (area = 5.59%). (**D**) Ascorbic acid 2,6-dihexadecanoate (C38H68O8) (area = 0.95%).

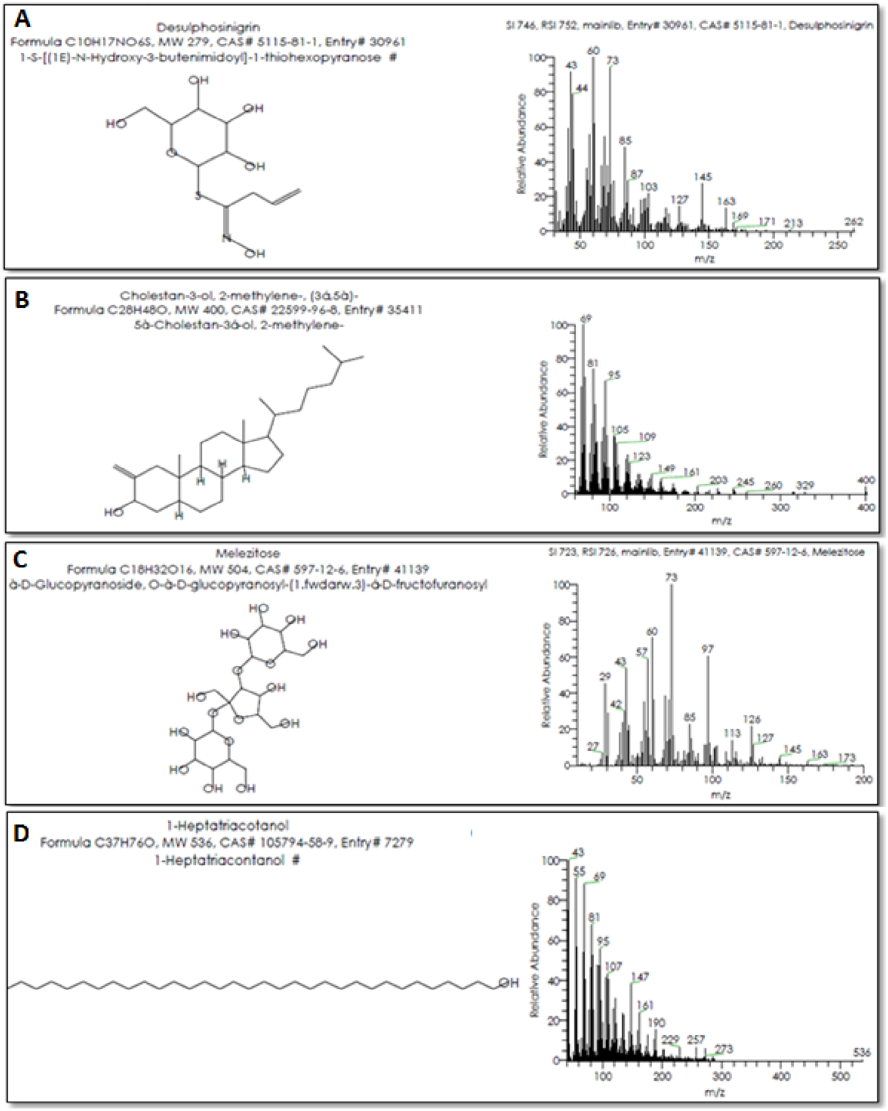

Figure S3. Chromatographic characteristics by GC-mass techniques showing the active principles in cacao bean meal. (**A**) Desulphosinigrin (C10H17NO6S) (area = 0.21%). (**B**) Cholestan-3-ol, 2-methylene (area = 0.17%). (**C**) Melezitose (C18H32O16) (area = 0.16%). (**D**) 1-Heptatriacotanol (C37H76O) (area = 0.17%).
